# Abdominal Section in the Country, with Report of Two Cases

**Published:** 1898-04

**Authors:** S. M. Jenkins

**Affiliations:** Summers’ Mill, Texas


For the Texas Medical Journal

### Abdominal Section in the Country, With Report of Two Cases.

BY S. M. JENKINS, M. D., SUMMERS' MILL, TEXAS.

[Read before the Central Texas Medical Association at Hillsboro, January 11th and 12th, 1897.]

*Gentlemen:*—What I shall say today about abdominal section will not be relative to a newer or improved method of making the incision or the procedure of exposing the offending part or organ; neither do I intend to advocate any particular technique of the disposal of the stump or severed pedicle, but would, did

I possess the knowledge, enter a plea for a better way of closing the abdominal wound than any we have today, for all our present methods leave the abdominal wall thinner along the line of incision, and thereby greatly increases the probability of a hernia.

My object in presenting this paper is simply to report two cases operated on by the writer during the past summer. These cases demonstrate the possibility of successful abdominal surgery even in the country, where we are deprived of the blessings and advantages of a hospital, and where we have the most inconvenient and unfavorable surroundings to contend with, and a clientele who have a holy horror of the surgeon's knife. But much of this can be overcome if the surgeon and his assistants are thoroughly alive to the necessity and importance of the minor details of aseptic surgery. I lay special stress on the observance of the minor or less important details, because they are more likely to be neglected than what is generally considered the more important ones. In my opinion, on the thoroughness with which these lesser details are carried out, in a large measure, depends the life or death of the patient.

Case 1.—W. R., male, aged about thirty years; diagnosis, chronic relapsing, appendicitis. On June 21, 1897, I did the operation, assisted by Drs. W. T. Davidson, Taylor Hudson, John R. Frazier and R. W. Smith. The patient's abdomen was shaved and well scrubbed with soap and warm water and bichloride solution the previous evening, and a clean towel pinned around him. Just before the operation he was scrubbed again with soap and warm water and solutions of permanganate of potassium and oxalic acid, ending with bichloride solution. The hands of the operator and his assistants were cleansed in like manner. Instruments, sutures and towels were thoroughly sterilized. An incision was made over McBurney's point and with some difficulty the appendix was found; it was twisted and curled upon itself and bound down with considerable adhesions, which were gently broken up. A silk ligature was passed around the appendix and secured. The peritoneum covering the appendix was divided and turned back a little, the appendix clipped off and the peritoneum was now brought forward over the stump and sewed together with silk. The abdominal cavity was well sponged out and the wound closed with silkworm gut. Dressings were now applied and held in place by a many-tailed bandage firmly adjusted to the figure. Patient rallied fairly well from the anæsthetic, but his temperature was sub-normal, so I gave him an enema of hot water and whiskey, placed hot bricks (which had been previously prepared) around him, and his temperature soon went up. He never once vomited. I redressed the wound on the eighth day; found no pus or suppuration. Stitches were removed on the twelfth day. Around two of the stitches were little stitch abscesses, from which oozed a drop or two of creamy pus. The stitch abscesses, I believe, were caused by the stitches remaining too long.

Some surgeons think there is a germ of infection in the deeper tissues which cannot be reached by antiseptics, and that this germ causes the stitch abscesses. They gave no trouble, however, and soon healed, as did the wound. Patient was up and walking about in the open air in about four weeks.

Case 2.—Mrs. B. W., aged 32, mother of one child, a girl, aged 11 years. Diagnosis of left ovarian cyst. August 5, 1897, I operated, assisted by Drs. W. T. Davidson and Taylor Hudson. The abdomen and pubes were well shaved and scrubbed, and were disinfected as in the preceding case. The hands of operator and assistants were well scrubbed with soap and warm water, using a new nail brush. Afterwards they were scrubbed with solutions of permanganate of potash, oxalic acid and bichloride of mercury. A free incision was made along the median line, and the cyst found to be about the size of a large orange and bound down by numerous adhesions. These were carefully broken up, and the oozing of blood controlled by compresses of hot towels. A needle carrying a double thread of strong braided silk was passed through the pedicle and the ends tied around either side. A slight oozing was now discovered coming from the broken adhesions. This was promptly secured by ligature and the pedicle divided. The right ovary being examined, was found slightly enlarged and cystic. It was promptly removed the same way that the left one was.

The cavity was well sponged out. A portion of the intestine that had been gently lifted out of the abdominal cavity and kept wrapped in warm towels was now returned to its natural place and the wound closed with silk worm gut sutures. Dressings were now applied and held in place by a firm many-tailed bandage. This lady was very weak and delicate to begin with, and her temperature and heart action were now both below normal. I gave her enema of hot water and whiskey, hypodermic of strychnine, and applied hot bricks. Her condition gradually improved, but as soon as she was from under the influence of the anesthetic she began to vomit, and kept it up pretty constantly for three and a half days in spite of all that could be done. I gave nutrient enemas of egg and whiskey at regular intervals. She complained of considerable pain, and I very reluctantly gave one-eighth grain of morphine, hypodermically, about twelve hours after the operation. This was all the morphine she had. I redressed the wound on the 8th day. It was perfectly clean and sweet; stitches were removed on the 10th day, at which time union was good. She sat up in bed on the 14th day, and was out of bed on the 20th day. The wound gave no trouble. She sat up, and walked about the house, but did not gain flesh and strength very fast, and her appetite was not good. I prescribed tr. nux. vomica, pepsin, Fowler's solution, and the mineral acids, and with the coming of cool weather she began to gain flesh and strength, and today enjoys good health. She is bright and cheerful, and says she is satisfied with her condition.

For the thorough surgical cleanliness in the preparation for these two operations, under unfavorable surroundings, great credit is due my esteemed friend, Dr. W. T. Davidson, of Belton, Texas.

